# Editorial: The One Health approach in the context of public health

**DOI:** 10.3389/fpubh.2024.1353709

**Published:** 2024-03-25

**Authors:** Sandul Yasobant, Shahzad Ali, Deepak Saxena, Daniela Patricia Figueroa, Mohiuddin Md. Taimur Khan

**Affiliations:** ^1^Center for One Health Education, Research and Development, and Department of Public Health Sciences, India Institute of Public Health, Gandhinagar, India; ^2^School of Epidemiology and Public Health, Datta Meghe Institute of Medical Sciences, Wardha, India; ^3^Global Health, Institute for Hygiene and Public Health, University Hospital Bonn, Bonn, Germany; ^4^University of Veterinary and Animal Sciences, Lahore, Pakistan; ^5^Faculty of Natural Resources and Veterinary Medicine, Santo Tomás University, Santiago, Chile; ^6^Department of Civil and Environmental Engineering, Washington State University, Tri Cities, WA, United States; ^7^Center for Molecular Discovery and Cancer Center, University of New Mexico, Albuquerque, NM, United States

**Keywords:** One Health, pathogen transmission, microbial resistance genes, antimicrobial stewardship, environmental protection and remediation, water-sanitation-hygiene, sustainable development

One Health recognizes the interdependence of the health of humans, domestic and wild animals, plants, and environmental ecosystems (1). Frontiers in Public Health recently published 11 articles on this Research Topic representing different aspects of this broad field. This approach mobilizes multiple sectors, disciplines, and communities at varying levels of society to work together, foster wellbeing and tackle threats. It also addresses the collective need for clean water-energy-air, safe and nutritious food, appropriate action on climate changes, and contributing to sustainable development. One Health (whose concepts date at least to the nineteenth century) is not new, but its application is increasingly critical. Our planet is being relentlessly altered by changes in climate and land use, including deforestation, industrialization, and intensive farming practices. Close contact with animals and their environments allows diseases to transmit from animals to humans. Disruptions in environmental conditions and habitats can also provide new pathways for diseases to pass to animals. The mobilization of people, animals, and animal products has increased significantly due to international trade allowing diseases to spread rapidly across the borders and around the globe. It is essential to interconnect these disciplines from regional to global levels, which will establish the pathway to meet the 17 sustainable development goals (SDGs) addressing these public health issues by 2030.

Some serological evidence were observed by several researchers regarding the transmission of viruses from animals to humans with minimal adaptation, but the interface between animals and humans is not much evident (2). Furthermore, human noroviruses have been detected from animal stool samples (3), and using the animal model experimental settings, the cross-species barrier capability was also confirmed by De Graaf et al. (4). The Global Consortium on Climate and Health Education surveyed 160 institutions to understand the stages of climate-health curricula for health professions. Shea et al. (5) concluded that the educational programs vary considerably between institutions and that most responders faced relevant challenges when trying to implement curricular changes in their institutions. Numerous processes were utilized to interlink the environment and human health, and coordinate with unknown threats like climate crisis, environmental pollution, and lack of biodiversity (6). O'Callaghan-Gordo et al. outlined the elements of an online Master's degree in planetary health, complementing a large number of existing post graduate courses in One Health. They also discussed the risk of confusion arising via the proliferation of subtly different approaches to health in the Anthropocene (a proposed name for our new era, in which humans are recognized as a force of planetary transformation).

Knowledge of the diversity and routes of transmission of pathogens is constantly expanding. For example, noroviruses are highly contagious, and infect a wide range of target but not limited to livestock animals, pets, marine mammals, rodents, and humans (7). Furthermore, a low concentration (10–18 virus particles) of this virus can cause severe infection (8). Zheng et al. (9) and Alam et al. (10) demonstrated that the human infections are mostly caused by GI, GII, GIV, GVIII, and GIX, although GII is the frequently detected genogroup worldwide. Using the One Health principles, Yasir et al. also identified the norovirus associated risk factors. The prevalence data reported by Yasir et al. was within the range of other study areas with similar climate, regional conditions, and food habits. Bovine norovirus has been identified as one of the possible etiologies of calf diarrhea (11) causing severe life-threatening effects on the neonatal calf (12). Due to the ever-increasing trade in the livestock industry, new and existing diseases are challenging for the industries. In addition to the microbial threat to the risk factors, Aslam et al. addressed that poor sanitation, unsanitary slaughterhouses, transportation of animals, nomadic lifestyle, and unskilled trained livestock and medical care professionals could be possible parameters to these risk factors and causes for spreading the Crimean-Congo hemorrhagic fever (CCHF). They also identified that the virus pathogenesis could be linked to either destroying cells directly through proliferation or damaging cells indirectly through the release of cytotoxic chemicals.

In separate studies, Ali and Alsayeqh and Bintsis (13) focused on pathogens such as *Escherichia coli, Salmonella, Campylobacter* (Figure 1), *Shigella, Listeria monocytogenes*, and others as well as toxins produced by *Staphylococcus aureus, Bacillus cereus*, and *Clostridium* species with an emphasis on their animal sources and prevention measures. These pathogens can cause gastrointestinal diseases with a focus on the mechanisms of toxigenic and invasive diarrhea (15). A need for a comprehensive approach to prevent and manage meat-borne diseases from production to consumption was identified by González et al. (16), which is consistent with a One Health approach, recognizing the interconnection between human, animal and environmental health (17). Furthermore, the aerosol transmissibility of intracellular pathogen *Coxiella burnetii* can cause Q fever, a meat-sourced bacterial infection, whose disease burden has been greatly lessened by a commercially available vaccine (18).

**Figure 1:**
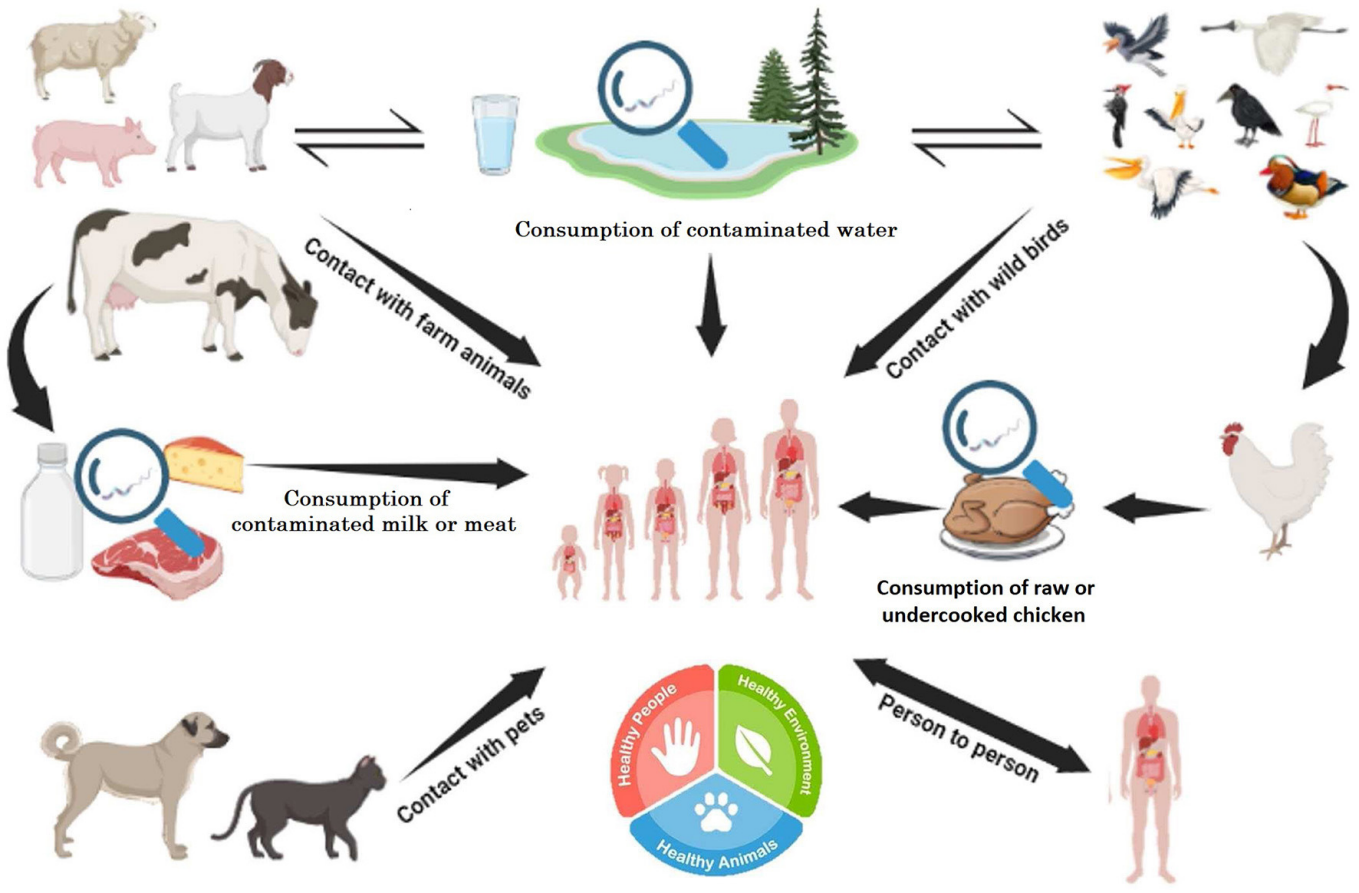
Reservoir and transmission of *Campylobacter*. *Campylobacter jejuni* can survive in various animals (poultry, cattle, and wild birds) and water reservoirs. Humans can acquire *C. jejuni* from consuming contaminated meat or meat products [(14); Ali and Alsayeqh].

The diversity of toxins, microbial resistance genes, and infectious diseases is extremely challenging to deal with because of their propagation natures. Scientists and project administrations are facing numerous complex challenges implementing a sustainable and effective antimicrobial stewardship (AMS) program; furthermore, it is essential to understand these challenges to tailor the stewardship initiatives according to the unique requirements (19), as discussed above. Hassan et al. identified several key issues that impede the implementation of the AMS program in their study areas. Those deficiencies led to well-structured monitoring, evaluation processes, and feedback provision below their standard limits. The World Health Organization (WHO) urged the healthcare organizations in developing and underdeveloped communities to conduct strategic planning and implementation toward the antimicrobial resistance action plan to standardize and overcome the challenges during the implementation of the AMS program. Pattnaik et al. performed an 86-item questionnaire to collect data consisting of semi-open questions based on socio-demographic characteristics, antibiotics usage, awareness of antimicrobial resistance, and healthcare utilization and quality of life (WHO-QOL BREF scale) in India. The findings from their study are very similar to those of Jordan and Nepal. It is essential to have knowledge on the proper antimicrobial use. Nearly 1/7th of the participants preferred taking antimicrobial medicines without prescription over the counter or without the consultation with healthcare workers, which is a very common scenario among the consumers in most of the developing, and underdeveloped countries.

Under the umbrella of One Health, individual, population, and ecosystem health are interconnected. To apply a species-spanning approach to medicine in One Health initiative, it is essential to incorporate not only contagious (infections) or other types of diseases (e.g., cancer or metabolic diseases), but also mental health and anything from behavioral problems related to addictions or depressions (20). Yun et al. identified that sleep deprivation may be one of the crucial factors governing the association of quick return (QR) to work with sleep disturbances (SD) and depressive symptoms (DS). The QR could shorten the duration of sleep, and cause disrupted and restless sleep (most found in the shift-work system) (21, 22). Yun et al. identified in their mediation analysis regardless of demographic factors and the working environment that the SD originating from QR could form a mediator associating QR and depression, which indicates that QR and DS are associated with each other. Following the One Health approach, numerous studies have been conducted to isolate and identify the proper drug molecules from natural sources to cure simple to complex diseases.

*Arisaema jacquemontii* Blume, a medicinal plant, treats several life-threatening diseases including viral infections because of its antioxidant, anti-cancerous, antimalarial, anti-vermicidal, and antiviral properties. Shehzadi et al. isolated and characterized (molecular docking) an antiviral COVID-19 protein [protease (6LU7)], from *Arisaema jacquemontii* Blume which has a maximum binding affinity of 8.1 kJ/mol and hydrogen bonding interactions. This group successfully concluded that this molecule (6LU7) could be useful to develop more phytochemical-based COVID-19 therapeutics. Rather and Mohammad (23) and Yang et al. (24) reported on several plant derived active ingredients including carotenoids, sterols, aliphatic compounds, monoterpenes and sesquiterpenes, triterpenoids, and others effectively. The plant-originated chemicals contain secondary metabolites, which could be effective drug molecules with several promising properties, e.g., antimicrobial effects, antioxidant activities, decreased platelets, detoxification of enzymes for modulation, anticancer properties, and hormonal modulation (25). These effective drug molecules block the replication in maturity of virus in the host body.

It is essential that clinical solutions to prevent diseases should be the last choice. For example, contaminated hands can also transmit the virus to people, fomites and other surfaces, potentially allowing complex additional transmission during any pandemic or epidemic. The elements of safe water, sanitation, and hygiene (WASH) can also limit transmissible disease transmission, including of COVID-19, though reducing respiratory spread is the most important concern (26). Other preventive measures for COVID-19 include isolation, quarantine, physical distancing (including lockdown), and personal protective equipment, including masks (27, 28). Khatib et al. identified through their reviews that the timely assessment and implementation of WASH along with other public health mediates could be essential ensuring acceptable preventive measures.

Environmental protection and remediation are the next concerns after the public safety in One Health mission. Ethylenediaminetetraacetic acid (EDTA) is widely used as an effective chelator for removing hazardous metals from soil (29) and other studies used phytoremediation for metals with or without using EDTA as a phytochelator, or specific types of plants/microbes as phytoremediators (30, 31). They concluded that EDTA could boost the metal uptake capacity. In a separate study, Ejaz et al. identified that Pb and Cd concentrations were significantly higher in shoots of *P. hysterophorus* plants than the roots regardless of EDTA during the treatment processes. However, the presence of EDTA enhanced the accumulation of cations K^+^, Na^+^, and Ca^+2^ in *P. hysterophorus*. The presence of EDTA increased further accumulation capacity of Pb and Cd on *P. hysterophorus* in soil. Chen et al. (32) demonstrated that phytoextraction of heavy metals from soil was enhanced by the high-biomass plants in presence of concentrated chelate-solubilized materials. These observations verified the importance of natural remediation process and its enhancement in presence of chelator. The plant ecosystem has been contributing significantly to remediation, drug molecule sources, greenhouse gas (GHG) minimization, carbon sequestration, air quality, human health, and others.

Carbon sequestration is the process of capturing and storing atmospheric carbon in the biosphere, mostly determined by the biotic resoluteness of plants including tree diameter, density, and probably stand basal areas (33, 34). Nagendra and Ostrom (35) noticed that the individual tree height is not associated with its diameter, but Feldpausch et al. (36) addressed the correlation between the height-diameter ratio and genetic nature of the species. The intraspecific competition, and abiotic drivers like precipitation, temperature, and soil types also play significant role in the sequestration process (37). Elevation is inversely proportional to the tree height (38); however, in the past the researchers used diameter at breast height (DBH) data of the tree ensuring the progress of and estimating carbon sequestration. Recently, tree height data has been used instead of DBH for more precise estimation of carbon (39). In addition, Ali et al. identified abiotic factors that mainly determined carbon sequestration in forest ecosystems along with the elevation gradients. They used a structural equation modeling approach to test a hypothesized causal relationship among the response variables using data from 200 plots covering sub-tropical thorn forests, sub-tropical broad-leaved forests, moist temperate mix forests, dry temperate conifer forests, and dry temperate *Pinus gerardiana* (*Chilgoza*) forest plots. They also quantified the effects of various forest types on carbon dioxide reduction leading to improved air quality. Furthermore, Potter and Woodall (40) concluded that species richness (SR) is also essential but not the most critical and appropriate metric in biodiversity and carbon sequestration.

One Health is a collaborative, multisectoral, and transdisciplinary approach working at the local, regional, national, and global levels. This editorial article briefly summarized 11 interesting studies on antimicrobial-resistant germs and pathogenicity, vector-borne diseases, contamination of the environment, carbon sequestration, antiviral COVID-19 protein, mobilization of worker behavior, propagation of tick-borne viruses, planetary health education framework, and human-animal interactions. The discussions in this edition categorically addressed how the One Health approach can prevent outbreaks of zoonotic disease, improve food safety and security, improve human and animal health by reducing antimicrobial-resistant infections, secure global health, and climate security, and protect biodiversity. We identified that the implementation of the One Health approach is a critical concern, and the possibility to meet the 17 SDGs by 2030 is questionable. Therefore, it is essential to have a more robust database with proper enforcement and regulatory strategies, organized policymakers, coordination of law makers, non-government organizations and stakeholders, and continuous global monitoring to expedite the implementation processes.

## Author contributions

SY: Formal analysis, Validation, Visualization, Writing – review & editing. SA: Formal analysis, Validation, Visualization, Writing – review & editing. DS: Formal analysis, Validation, Writing – review & editing. DPF: Formal analysis, Validation, Visualization, Writing – review & editing. MMTK: Conceptualization, Data curation, Formal analysis, Investigation, Methodology, Resources, Supervision, Validation, Visualization, Writing – original draft, Writing – review & editing.
